# Evaluation of Quality of Life and Satisfaction of Patients with Neuropathic Pain and Breakthrough Pain: Economic Impact Based on Quality of Life

**DOI:** 10.1155/2018/5394021

**Published:** 2018-09-05

**Authors:** María Candelas Madariaga Muñoz, Francisco Villegas Estévez, Antonio Javier Jiménez López, Ana Cabezón Álvarez, Begoña Soler López

**Affiliations:** ^1^Hospital Infanta Sofía, San Sebastián de los Reyes, Madrid, Spain; ^2^Consorcio Hospitalario Provincial de Castellón, Pain Unit, Castellón, Spain; ^3^Kyowa Kirin Farmacéutica, S.L.U., Medical Department, Madrid, Spain; ^4^E-C-BIO, S.L., Medical Department, Las Rozas, Madrid, Spain

## Abstract

**Objective:**

The study objective was to assess the quality of life and satisfaction with treatment of patients with chronic neuropathic pain (CNP) who experience breakthrough pain (BTP) and to assess its economic impact.

**Design:**

Cross-sectional observational study.

**Setting:**

Fifteen pain units from Spanish hospitals completed the study.

**Participants:**

A total of 124 patients with adequately controlled CNP who experienced BTP were enrolled into the study.

**Intervention:**

No interventions were required.

**Main Outcome Measures:**

Quality of life was assessed using the SF12 v2 questionnaire, the results of which were used to calculate the estimated costs per patient and month and the SF-6D Health Utility Index. Patient satisfaction with treatment received for CNP and for BTP was assessed using a 10-point visual analogue scale. Other associated symptoms were analyzed using the ESAS (Edmonton Symptom Assessment System).

**Results:**

Patients had a mean age of 60.2 years (95% CI 58.4-63.3), and 46.8% (58) were males. 18.9% (23) experienced their first episode of BTP. A severe impairment of the physical component of SF12v2 was noted, with 94% of patients below the mean score of the population, while 88% had values lower than normal for the mental component. Mean cost per patient and month was $679 and was significantly greater in males ($763 versus $606), 4.96 times greater than in healthy population, and approximately double the cost of patients with CNP in Spain.

**Conclusions:**

Occurrence of BTP in patients with CNP causes a substantial increase in healthcare costs which is significantly greater in older males.

## 1. Introduction

The International Association for the Study of Pain (IASP) defines neuropathic pain as pain initiated or caused by a primary lesion or dysfunction of the peripheral or central nervous system. Such lesions include a large number of neurological conditions including diabetic polyneuropathy and other sensory neuropathies, trigeminal neuralgia, postherpetic neuralgia, stroke, spinal cord lesions, and multiple sclerosis, as well as other more common conditions such as lumbar and cervical radiculopathies and traumatic or postoperative injuries [1–4] Neuropathic pain has a high prevalence in the general population of 5% to 8% [5–7] and is usually difficult to control in clinical practice, because patients have a poor response to treatment. It has been reported that, regardless of the treatment option used, adequate analgesia is achieved in less than half of the patients [8–12].

A high proportion of patients with neuropathic pain experience breakthrough pain (BTP) at some time during the course of their condition. BTP is defined as a “transient exacerbation of pain that occurs either spontaneously or in relation to a specific predictable or unpredictable trigger, despite relatively stable and adequately controlled background pain” [13]. Incidence of BTP ranges from 23% and 93% depending on whether or not the patient has cancer, on the progression status of the tumor and on the clinical setting where it is determined [14].

Occurrence of BTP has a very significant impact on the quality of life of patients and is also characterized by being associated with a high use of healthcare resources [15, 16]. Patients with neuropathic pain have a markedly poorer health-related quality of life than the general population, which results in up to a threefold increase in their direct healthcare costs [17]. But association of neuropathic pain to BTP is even more harmful for their quality of life [16]. Assessment of quality of life impairment may serve as an indirect measure of the economic impact of the disease. Tools such as the SF-12 quality of life questionnaire can relate quality of life of the patient to cost of the affected patient and compare this cost to that of the general population of the same age and sex [18].

The quality of life impairment experienced by patients with neuropathic pain who also suffer BTP, the medical costs in such patients, and their degree of satisfaction with treatment they receive for the two types of pain are currently unknown and were the primary objectives of the study.

## 2. Methods

### 2.1. Study Design and Ethical Standards

A retrospective observational study was designed. The ethics committee of Hospital Universitario La Paz in Madrid (Spain) approved the study (17 March 2016). The basic ethical principles of the Declaration of Helsinki were respected in the study.

Fifteen pain units from 13 Spanish provinces participated, enrolling patients from June to December 2016.

### 2.2. Selection Criteria

Patients of both sexes over 18 years of age with history of neuropathic pain, adequately controlled at the time of selection, as shown by a score of four points or less in a 10-point visual analogue scale of pain intensity, this being the maximum pain value, were enrolled. Background pain was considered neuropathic in nature if the score in the DN4 questionnaire (Dolour Neuropathique 4 questions) was four points or greater [19, 20]. Patients should have BTP fulfilling the Portenoy criteria [21], for which they could or could not be receiving treatment. Patients with cognitive impairment or severely affected by their background disease, expected to be noncollaborative, or who would not be able to complete the study questionnaires were excluded.

All patients were given written information about the study and its objectives and signed an informed consent before study entry.

Patients were consecutively selected among the patients who attended the outpatient clinics of pain units. No specific treatment was required to be included in the study.

### 2.3. Quality of Life

The primary objective, assessment of quality of life of patients, was evaluated with the SF-12 questionnaire at the time of the visit. The SF-12 questionnaire is a short version of the SF-36 Health Survey. It is a self-administered questionnaire that measures health-related quality of life from the patient perspective and is completed in approximately two minutes. SF-12 collects 12 questions to the patient in Likert scales of 3 to 5 responses. The SF-12 has two components, physical component (PC) and a mental component (MC) with the following dimensions: physical component: PF: Physical Functioning; RP: Role Physical; BP: Bodily Pain; GH: General Health; and mental component: VT: Vitality; SF: Social Functioning; RE: Role Emotional; MH: Mental Health [22].

The results of the SF-12 questionnaire allowed the calculation of estimated costs per patient and month, evaluation of costs by age group and sex, and comparison of such costs to those for a healthy patient of the same age group and sex [18]. The scores of the physical and mental components of the SF12-v2, their eight dimensions, and the costs per patient and month were calculated using specific licensed software of the questionnaire owner (QualityMetric Health Outcomes™ Scoring Software 4.5) [22]. The normal population values used for the comparisons were those of the United States of America (USA), as it has been reported that no significant differences exist between countries in normal values [23]. Differences less than 0.2 times the standard deviation were considered small, those around 0.5 would be moderate, and those 0.8 times or higher would be great. A patient was considered at risk of depression when the mental component score (MCS) of the SF-12v2 was 42 points or less. This cut-off value was the result of comparison of the MCS scores with a short form of CESD (Center of Epidemiologic Studies Depression scale) and the NIMH (National Institute of Mental Health) Diagnostic Interview Schedule (DIS) criteria for clinical depression. Sensitivity and specificity across the full range of MCS scores was evaluated using receiver operating characteristic analysis and the cut-off for the MCS was scores of 42 [22, 24].

The SF-6D is a health index derived from the SF-12 v2 for use in economic evaluation studies. Seven of the eight domains of SF-12 v2 were used to calculate SF-6D. The score ranges from 0.0 (worst health status/dead) and 1.0 (best health status). Medical expenditure is calculated using a regression equation that considers health-related quality of life data, sex, and age. The algorithm to calculate medical costs is validated and uses data from the Medical Expenditure Panel Survey, a representative national survey on use of healthcare resources and expenditure in noninstitutionalized US population [25]. Specific licensed software of the questionnaire owner provides the costs results (QualityMetric Health Outcomes™ Scoring Software 4.5) [22].

### 2.4. Patient Satisfaction

Patient satisfaction with treatment received for background pain and BTP at the time of the visit was assessed using a 0-10-point visual analogue scale (VAS), where 0 represented “Not at all satisfied” and 10 “Very satisfied”.

As a secondary objective, an analysis was made of control of symptoms associated with the disease, assessed using visual analogue scales (Edmonton Symptom Assessment System, ESAS) that rated pain, tiredness, nausea, depression, anxiety, drowsiness, anorexia, discomfort, dyspnea at rest, and insomnia in the past 24 hours [26]. Symptom control was considered to be adequate when symptom scores were four points or less in a 10-point scale where a value of 10 represented the greatest symptom intensity. A patient was considered to be adequately controlled when score was four points or less in all 10 categories.

Information was collected on age, sex, and socioeconomic status (low, middle, or high), on patient weight and height, and on disease history. The characteristics of background pain, the DN4 score, characteristics of the BTP listed in Table 1, the mean daily number of episodes, and mean duration of the BTP episodes were recorded. Information on the treatments that patients were receiving for neuropathic pain and BTP and information on other treatments administered for associated diseases were collected.

### 2.5. Sample Size Calculation

The primary study variable was assessment of quality of life using the SF-12 questionnaire at the time of the visit and comparison of the scores to population standards. The normal value of the physical component of the SF-12 questionnaire for subjects aged 55-64 years in Spain is estimated to be 46.97 points, with a standard deviation of 10.21 [27]. Assuming the null hypothesis that there were no differences in the scores of the physical component of the SF-12 questionnaire between study subjects and the population standard for subjects of the same age, with a two-sided significance level (alpha) of 0.05, a sample of 124 patients had a power of 80.4% and a precision of 1.81 points.

### 2.6. Statistical Analysis

A descriptive analysis was performed of study variables. Frequencies and percentages were calculated for qualitative variables, and mean, standard deviation, 95% confidence intervals, and minimum and maximum were calculated for continuous variables.

Comparisons between qualitative variables were made using a Fisher test or a Chi-squared test. Student's t-test was used to compare independent groups in the case of quantitative variables.

When differences in quality of life and costs were assessed using different control variables, the factorial model of analysis of variance was used, applying Bonferroni or Games-Howell correction depending on homogeneity of variance, to control error for multiple comparisons. For analysis of the SF-12 questionnaire, specific software from questionnaire owners was used [22]. Significance level was set at 0.05. SPSS version 14.0 was used for the statistical analysis.

## 3. Results

A total of 124 patients who met all selection criteria were enrolled into the study. Of these, 53.2% (66) were females and 46.8% (58) were males, with a mean age of 60.8 years (95% CI 58.4-63.3), a median age of 60 years, and ages ranging from 34 and 85 years. Females were significantly older (p=0.016), with a difference of 5.9 years (95% CI 1.1-10.7).

Of all subjects, 68.5% (85) had a middle socioeconomic status, 22.6% (28) a low status, and 8.9% (11) a high socioeconomic status. Mean body mass index was 26.2 kg/m^2^ (95% CI 25.4-27), with no differences between males and females (p=0.285).

History of oncological disease was found in 37.1% of study patients (46). Gastrointestinal tumors were most common (23.9%; n=11), followed by lung (21.7%; n=10), breast (10.9%; n=5), prostate (8.7%; n=4), and other tumors (34.8%; n=16). Mean time from tumor diagnosis to the study visit was 2.7 years (95% CI 2-3.4), with a median of two years (range, 0.21-9 years).

The main cause of chronic pain was spine problems (35%; 43 patients), followed by tumors (27.6%; 34 patients) and peripheral neuropathy (19.5%; 24 patients).

Mean score in the DN4 scale for assessment of neuropathic pain was 6.1 (95% CI 5.8-6.5), with a median of 6 points.

### 3.1. Characteristics of BTP

Patients had the first episode of BTP in 18.9% (23) of the cases and were on follow-up of the remaining 81.1% (99) with 2 missing data.

Table 1 shows the characteristics of BTP observed in study patients. The mean daily number of episodes of BTP was 3.2 (95% CI 2.8-3.6), and episodes had a mean duration of 40.6 minutes (95% CI 32.4-48.7).

A single active ingredient was administered to 112 patients (90.3%) to treat BTP, while seven patients (5.7%) received no active ingredients and five patients (4%) were administered two active ingredients.  Table 2 shows the active ingredients used to treat BTP. The most frequent was fentanyl, 70.9% (61 patients) using the in the nasal spray administration, 23.3% (20) sublingual, and 5.8% (5) the inhaled route.

### 3.2. SF-12 v2 Quality of Life Questionnaire

All SF-12 v2 items were completed by 97.7% of patients, with 100% validity for convergence and discriminant analysis. Calculations of the physical and mental components were obtained from the data of 122 of the 124 patients enrolled.

**Figure 1:**
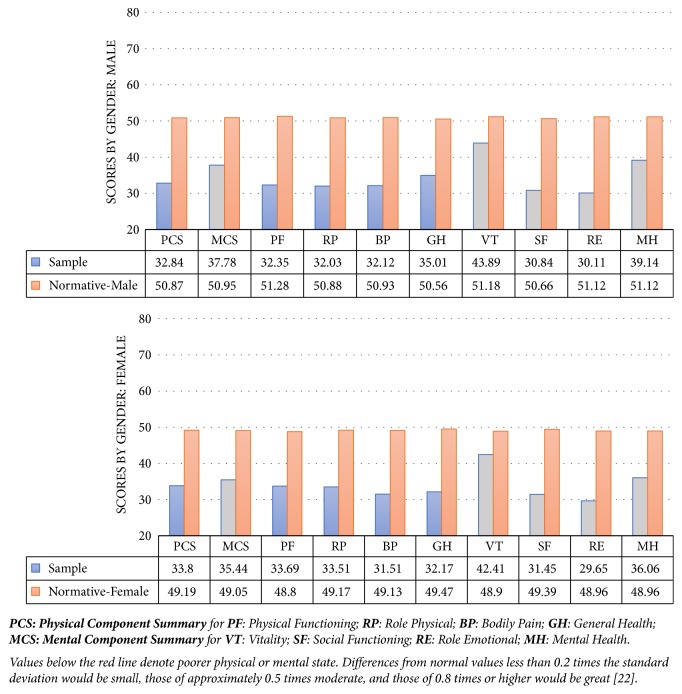
Scores of the physical and mental components of the SF-12 v2 quality of life questionnaire and their domains by patient sex and comparison to the normal reference values in healthy populations.

Figure 1 shows the results of the physical and mental components of the SF-12 v2 quality of life questionnaire and its respective domains by sex. A severe impairment in the physical and mental components was observed in patients as compared to the population means. Differences between males and females in the** general health** dimension were statistically significant (p=0.044), with 2.8 points less in females (95% CI 0.1-5.6). Scores in the** mental health** dimension were also lower in females, with differences of 3.1 points (95% CI 0.3-5.8), p=0.028. A univariate analysis found no significant differences in the components and dimensions of the SF-12 v2 questionnaire by sex, between patients with and without tumors, by socioeconomic status, or depending on whether the patient had experienced the first episode of BTP or was on follow-up.

In the multivariate analysis including data of 120 patients, where the scores of the physical or mental component of the SF-12 v2 questionnaire were the dependent variable, the independent variables, sex, age, socioeconomic status, patient with BTP de novo or under follow-up, and patient with and without tumor, resulting were not statistically significant.

In the physical component, 94% of patients in the study were below the mean score of the population, 5% within the normal range, and 1% above normal values. In the mental component, 88% were below the normal levels of the general population, 11% were within normal values, and 1% were above normal values. There was a greater proportion of males with physical component scores below normal values (98% versus 91%) and a greater proportion of females with mental component scores below normal values (89% versus 86%).

Seventy-two percent of patients enrolled into the study were at risk of depression, with a greater proportion of females (80% versus 64%), p<0.05.

### 3.3. Medical Expenditure and Health Utility Index (SF-6D)

**Figure 2:**
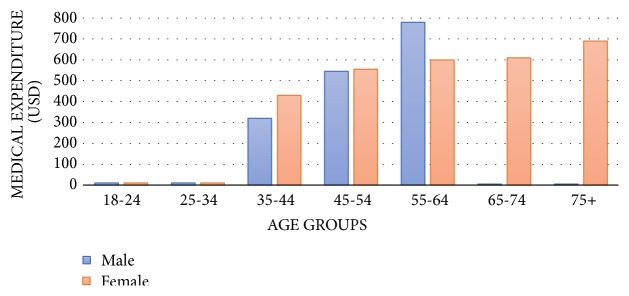
Estimation of monthly cost per patient (USD) as determined from the SF-12 v2 quality of life questionnaire by age and sex groups.

Mean monthly cost of each patient participating in the study was $679.24 (95% CI 622.3-736.2), resulting in females of $605.7 (95% CI 553.7-657.6) and in males of $763.2 (95% CI 658.9-867.4). There were** statistically significant differences in medical expenditure between males and females (p=0.006), with a cost $157.5 greater in males (95**%** CI 46.5-268.5). **In Figure 2 the monthly cost per patient by sex and age group is shown.

The overall study sample had a score of 0.51 (95% CI 0.50-0.53) in the Health Utility Index.

Univariate analysis showed no statistically significant differences in medical expenditure or the utility index between patients with and without tumors, between patients with BTP de novo or on follow-up, or by socioeconomic status and age. No sex differences were seen in the utility index.

In the multivariate analysis including data of 120 patients, including the control variables listed in the previous paragraph, no significant relationship was seen with the utility index, but patient sex and age were found to be statistically significant for monthly medical expenditure (p<0.0001 and p<0.0001, respectively). Costs were significantly greater in males ($235.22; 95%CI 137.92-332.52) and are greater if the patient is older ($12.64 per year; 95% CI 9.116.19). Age/sex interaction was statistically significant (p<0.0001). Costs were greater in females until 49 years of age and in males after that age.

### 3.4. Patient Satisfaction with Treatment

Ninety-six patients (77.4%) were satisfied with treatment for chronic pain, 66.4% (81 patients) were satisfied with treatment for BTP, and 56.6% (69 patients) were satisfied with both treatments.

### 3.5. Assessment of Control of Symptoms Associated with the Disease (ESAS)

**Figure 3:**
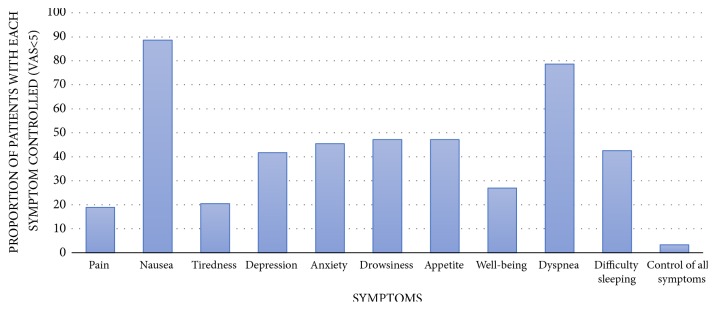
Proportion of patients with adequate symptom control, with scores lower than 5 points in a 10-point visual analogue scale.

Figure 3 shows the proportion of patients with adequate control of each of the symptoms assessed by the ESAS. Only four patients (3.3%) had adequate control of all 10 symptoms assessed.

## 4. Discussion

Patients with neuropathic pain are a group who attend pain units very often. The frequency with which these patients experience BTP is also high. Assessment of the quality of life of study patients with background neuropathic pain and BTP showed a severe impairment of the physical component of the SF-12 v2 quality of life questionnaire and a great proportion of patients with quality of life values lower than normal standard in both the physical component (94%) and the mental component (88%). The SF-12 v2 questionnaire also revealed that 72% of patients were at risk of depression, as compared to 20% of the general population [27]. No relationship was found in the study between quality of life and socioeconomic status, but we think that future studies should consider the use of additional variables such as social isolation, commonly seen at the clinic in these patients and which may have an impact on their quality of life.

Mean healthcare cost calculated based on the SF-12 v2 questionnaire was $679, but cost was significantly greater in males and the older the age. As compared to the mean healthcare cost in the healthy US population of $137, in this study, with patients with CNP associated with BTP, medical expenditure was 396% higher than in the normal population that is 4.96 times greater than in the healthy population. Monthly cost per patient with neuropathic pain in Spain has been estimated at approximately €363, which is about half the cost seen in the study where the patient has both neuropathic pain and BTP [28]. This difference may be explained due to the way the costs have been calculated, based on US data, where per capita spending is higher than in Spain. Although there were no sex differences in the quality of life results, greater healthcare costs were seen in males older than 49 years. As the mean age of the selected patients who attended our clinics was 60.8 years, this was a group incurring high costs.

Opioids have been reported to be the main relief factor in BTP episodes in 44.61% of patients, while 26%-44% find relief avoiding the movement that causes pain, and 12%-20% do not appear to achieve adequate relief with drug and nondrug measures [21]. Because of the large etiological diversity of BTP, its treatment requires a multiple approach with drugs, especially opioids, being essential for the success of treatment. However, many patients (up to 77%) are not diagnosed or adequately treated [29].

The study assessed the degree of patient satisfaction with treatment received for CNP and BTP. Treatment satisfaction was reported 77.4% of patients with CNP and by 66.4% of patients with BTP. Single drug treatment was given to 90.3% of patients being fentanyl the most commonly used active ingredient: 70.9% (61 patients) using the nasal spray administration, 23.3% (20) sublingual, and 5.8% (5) the inhaled route. The lower degree of satisfaction with treatment for BTP may be related to the greater difficulty of such treatment in this group of patients, secondary to the greater difficulty in treatment of CNP, since the characteristics of BTP were similar to those reported in other studies [14].

The cross-sectional design of the study was adequate to assess the study objectives, with the limitations inherent to studies of a descriptive nature. The small number of missing data allowed for exploratory inferential analysis in 120 of the 124 patients.

## 5. Conclusions

The study conclusion is that occurrence of BTP in patients with CNP causes a substantial increase in healthcare costs which is significantly greater in older males.

## Figures and Tables

**Table 1:** Characteristics of BTP in study patients.

		N	%
Location of BTP	Head/face/mouth	14	11.3
	Neck	2	1.6
	Shoulder	1	0.8
	Arm	7	5.6
	Chest	15	12.1
	Abdomen	11	8.9
	Lumbar/sacral/coccygeal	41	33.1
	Leg	33	26.6
	Missing	0	

Onset of breakthrough pain	Gradual	44	36.1
	Sudden	78	63.9
	Missing	2	

Intensity of BTP	Mild	2	1.6
	Moderate	14	11.3
	Severe	78	62.9
	Unbearable	30	24.2
	Missing	0	

Does any event increase BTP?	No, it is spontaneous	67	54.5
	Yes, it is incidental	56	45.5
	Missing	1	

When does BTP occur?	At night	14	11.3
	In the daytime	52	41.9
	Unrelated	58	46.8
	Missing	0	

How does BTP occur?	It is unpredictable	79	64.2
	It is predictable	44	35.8
	Missing	1	

Type of BTP	Somatic	5	4.1
	Visceral	4	3.3
	Neuropathic	64	52
	Mixed	50	40.7
	Missing	1	

**Table 2:** Active ingredients administered to treat BTP.

	N	%
Acetylsalicylic acid	1	0.8
Bromazepam	1	0.8
Clonazepam	1	0.8
Dexketoprofen	2	1.6
Fentanyl	86	70.5
Gabapentin	1	0.8
Ibuprofen	1	0.8
Lidocaine	2	1.6
Metamizole	6	4.9
Morphine	4	3.3
Naproxen	1	0.8
Oxycodone	2	1.6
Acetaminophen/Caffeine/ Codeine	1	0.8
Pregabalin	1	0.8
Tramadol	8	6.6
Tramadol/Acetaminophen	4	3.3

Total	122	

## Data Availability

The database used to support the findings of this study is available from the corresponding author upon request.
